# Emergency department-based 3D echocardiogram use: a case series

**DOI:** 10.1186/s13089-020-00193-4

**Published:** 2020-09-30

**Authors:** Sajith Matthews, Phillip D. Levy

**Affiliations:** 1 Division of General Medicine, Department of Internal Medicine, Wayne State University, 4201 St. Antoine Street, UHC 5C, Detroit, MI 48201 USA; 2 Department of Emergency Medicine, Wayne State University, Detroit, USA

**Keywords:** Three-dimensional echocardiography, Emergency department, Acute care

## Abstract

**Background:**

Three-dimensional echocardiography (3DE) with real-time volumetric imaging can be a vital modality in clinical practice. Despite its potential, it remains underutilized in the acute care setting.

**Case presentation:**

We present two cases describing the use of 3DE in the emergency department (ED) for acute heart failure (AHF) and discuss the potential benefits of routine use in acute care settings.

**Conclusions:**

Three-dimensional echocardiography offers unique information as it relates to cardiac structure and function, and can be valuable for diagnosis and clinical decision-making in the ED.

## Background

Three-dimensional echocardiography (3DE) given its real-time quantifications is a valuable modality in clinical practice, yet its use has had little exploration in the acute care setting [1]. Given its superiority to two-dimensional echocardiography (2DE) in calculating left ventricular volumes and ejection fraction (EF) [2], there remains much potential for use in acute heart conditions. We present two cases describing the use of 3DE in the emergency department (ED) in acute heart failure (AHF), with images obtained prior to therapeutic intervention. We discuss the potential benefits of 3DE for such indications in the future, highlighting needs for scalability.

## Case presentation

Patient 1 was a 58-year-old man who presented to the ED with a 2-week history of worsening dyspnea on exertion accompanied by bilateral lower extremity edema. He had a history of regular cocaine use and a diagnosis of heart failure with reduced ejection fraction (HFrEF) with the last EF being 15% 1 month prior to his ED visit. He described a diet that was high in salt intake and alcohol use. Physical exam was notable for tachycardia, but had otherwise normal vitals. He had audible pulmonary crackles and an S3 gallop on auscultation. He also had 3+ pitting edema extending up to the knees and jugular venous pressure of 12 mm Hg. Laboratory results were notable for an N-terminal pro b-type natriuretic peptide (NT-pro BNP) of 5279 pg/mL. His troponin, electrolytes and creatinine were normal.

Patient 2 was a 53-year-old man with a history of HFrEF due to ischemic cardiomyopathy from a recent coronary angiogram, presenting with a 3-day history of dyspnea (at rest and exertion) and orthopnea. He reported no improvement after taking his home dose of oral furosemide. He had an EF of 15% four months prior to his ED visit. Physical exam was notable for stable vitals. He appeared to be mildly dyspneic and had bilateral crackles on auscultation. His JVP was 14 mmHg. Laboratory results revealed an NT-pro BNP of 1209 pg/mL. His troponin and electrolytes were normal. His chest X-ray showed cardiomegaly with pulmonary vascular congestion.

**Fig. 1:**
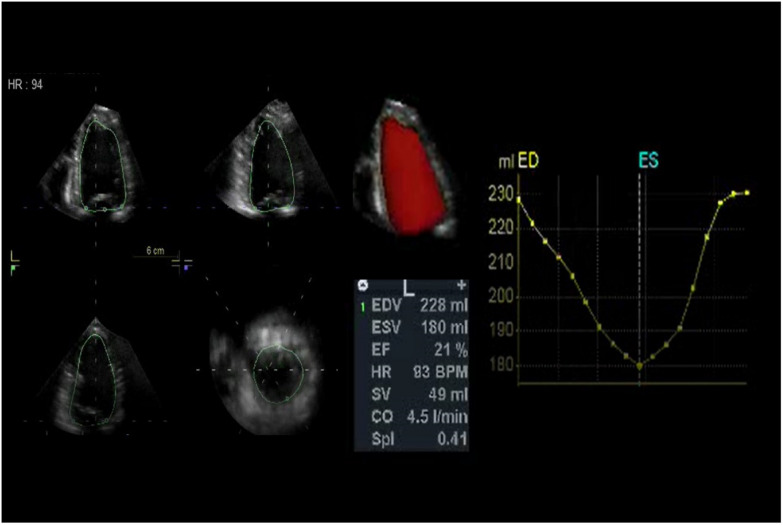
Graphic representation of LV volume, EF and sphericity index of patient 1

Please see Table 1 and Fig. 1 for the 3DE measurements.

**Table 1.** 3DE-derived left ventricular ejection fraction, sphericity index and left ventricular volumes

	EF (%) Normal (57–77%)	LVEDV (mL) Normal (106–214 mL)	LVESV (mL) Normal (26–82)	Stroke volume (mL) Normal (72–144 mL)	Sphericity index (SI) Normal < 0.25	Protocol time (min)
Patient 1	28	221	160	61	0.41	10 ± 2
Patient 2	21	228	180	48	0.50	10 ± 2

## Conclusions

The above cases are the first to appear in the literature demonstrating the use of 3DE in the ED. While use of 3DE in these cases was purely observational, the precision of LV volumes and EF obtained with such an approach may be helpful in the workup of AHF, particularly in patients with new onset of the diagnosis and a suspected ischemic component [3]. Prompt ischemic workup can be cost effective, where upfront testing leads to lower resource utilization and fewer hospitalizations [4]. With the advent of high-sensitivity cardiac troponins, AHF patients will have biomarker evidence of myocardial injury [5]. When combined with 3DE-derived LV volumes, it can be an effective noninvasive modality to help determine if such patients need an acute coronary intervention [3].

Why might this be? Along with volume, LV shape is a useful parameter to assess LV dysfunction induced by ischemia, with the ventricle becoming more globular as LV function deteriorates. The sphericity index (SI) with the use of 2DE has been used to quantify this change; however, 3DE represents ventricular shape more accurately and may have utility as an earlier, more accurate predictor of ischemic wall motion effects as it divides the LV end-diastolic volume by the volume of a sphere, the diameter of which is the LV major end-diastolic long axis [6]. In addition to SI being the most sensitive (100%) and specific (90%) indicator to early LV remodeling after myocardial infarction (MI) [6], it can be essential in the acute ischemic setting as regional and global sphericity indices are increased in patients with regional wall motion abnormalities (RWMA) and dilated cardiomyopathy [7]. These measurements also correlate well with 3DE-derived LV systolic dyssynchrony index (3D SDI) in patients with recent anterior MI (AMI) for LV remodeling, having superior incremental value in detecting changes in the LV (dilation often occurring within 3 h of AMI due to microvascular no reflow to the epicardium) [6, 8] over 3D volumetry or other 2D parameters [9]. Detecting such subtle changes with 3DSI and 3DSDI, which serve as independent predictors of LV remodeling, can be very helpful in determining which patients would benefit from a coronary intervention, especially when combined with troponin levels. The same study also showed 3DSI, 3DSDI, and LV volumes were obtained within 15–25 min, which is within 5–15 min of the time needed for our study. In contrast, 2DE has limitations as it relates the ratio of the cross-sectional area of the LV from an apical four-chamber view to a circle with a diameter equivalent to LV major end-diastolic long axis and thus is unable to account for discrete changes in regional LV shape. The net result could be misleading information regarding the acuity of findings, a circumstance that would have detrimental impact on decisions related to coronary intervention.

While just two cases, we demonstrate that 3DE can be performed in the acute care setting with an average scan time of 10–12 min. Further study is needed to establish the feasibility of routine use of 3DE in the ED and should include a comparison with other modalities that provide similar information such as cardiac MRI to determine accuracy as well as cost effectiveness. Ideally, the latter would account for potential treatment implications of 3DE, especially the directed use of interventions to reduce the adverse effect of acute ischemia on LV function.

## Data Availability

This manuscript adhered to Hospital policies regarding image collection and research. Data can be made available upon request.

## Abbreviations

3DE: Three-dimensional echocardiography
AHF: Acute heart failure
ED: Emergency department
